# An Internet-Based Cancer Clinical Trials Matching Resource

**DOI:** 10.2196/jmir.7.3.e24

**Published:** 2005-07-01

**Authors:** James M Metz, Carolyn Coyle, Courtney Hudson, Margaret Hampshire

**Affiliations:** ^2^EmergingMedNew York, NYUSA; ^1^University of Pennsylvania Cancer CenterPhiladelphia, PAUSA

**Keywords:** Internet, neoplasms, clinical trials

## Abstract

**Background:**

Many patients are now accessing the Internet to obtain cancer clinical trials information. However, services offering clinical trials recruitment information have not been well defined.

**Objectives:**

This study describes one of the first Web-based cancer clinical trials matching resources and the demographics of users who were successfully matched.

**Methods:**

OncoLink is the Internet-based educational resource managed by the University of Pennsylvania Cancer Center (UPCC) and serves between 1 and 2 million pages per month to over 385000 unique IP addresses. OncoLink launched one of the first clinical trials matching resources on the Internet that allowed patients to enter demographic data through a secure connection and be matched to clinical trials. For patients with matches to potential trials, appointments were facilitated with the principal investigators.

**Results:**

While we did not keep track of patients who could not be matched, 627 patients who submitted online applications between January 2002 and April 2003 were successfully matched for potential enrollment in clinical trials. The mean age of the patient population was 56 years (range 18–88 years). Males represented 60% of the patient population, and over 90% of users were Caucasian. Most of the applications were from patients with colorectal cancer (13%), lung cancer (14%), melanoma (10%), and non-Hodgkin's lymphoma (9%).

**Conclusions:**

This report shows that a significant number of patients are willing to use the Internet for enrolling in clinical trials. Care must be taken to reach patients from a variety of socioeconomic and racial backgrounds. This Internet resource helps to facilitate a consultation with a cancer patient who is prescreened and motivated to enroll in clinical trials.

## Introduction

### Clinical Trials Recruitment

Recruitment of cancer patients to clinical trials in the United States has remained stagnant for a number of years. Only 2% to 4% of all adult patients with newly diagnosed cancer participate in clinical trials [1]. Because of the low accrual rate, many clinical trials have lacked the power to make significant conclusions [2].

A host of physician-related and patient-related factors have led to the low rate of enrollment in clinical trials for adult cancers in the United States. The prevalent theme among studies examining the barriers to enrollment has been the lack of physician and patient awareness of open clinical trials and the unavailability of protocols to both the patient and physician at the time of consultation. Less than half of eligible patients are even considered for clinical trials [3-5]. Many physicians have concluded that approaching a patient about a clinical trial intrudes upon the patient-physician relationship [6-10]. Also, many physicians do not have the appropriate protocol available to them when evaluating a potentially eligible patient [3]. Patient-related factors that limit enrollment in clinical trials include absence of knowledge about clinical trials, unwillingness to be randomized to treatment, time constraints, distance from treatment center, insurance denial, and distrust of the medical establishment [3,7-12]. Once a clinical trial is offered to a patient, the overall rate of acceptance is 15% to 40% [1,6,11]. Thus, there is clear potential for improvement in clinical trials enrollment.

### Cancer Patients' Use of the Internet

Cancer patients are increasingly using the Internet as a source of medical information. This is a reflection of the overall availability of computers and access to the Internet. Forty to fifty percent of cancer patients use the Internet to search for information, and this number continues to rise [13-16]. Many are looking for information regarding cancer clinical trials [14,15]. The Internet is a resource that can rapidly reach a wide geographic population that otherwise may not have access to clinical trials information. However, there are concerns that minority and elderly patients are less familiar with and have limited access to the Internet [17,18]. Recently, a number of services have been offered over the Web to facilitate enrollment in clinical trials. The increasingly widespread use of the Internet makes it a potential source by which patients can become aware of and enroll in clinical trials, improving the rate of clinical trial enrollment.

### Internet-Based Clinical Trials Recruitment

Although a number of clinical trials services have arisen on the Internet over the past few years, data regarding these services are lacking. The first reported Web-based matching services for clinical trials appeared in the HIV community [19,20]. However, these services did not use complex algorithms because they were dealing with a single disease with a small number of variables. Previous Internet-based cancer clinical trials information has solely been listings of available trials or limited matching to a specific type of cancer [21-23]. This study describes the first broad Web-based matching service and the initial demographic parameters of the patients using the resource.

## Methods

### OncoLink's Clinical Trials Matching Resource

OncoLink (www.oncolink.org) is the oldest and one of the largest general cancer information resources on the World Wide Web. It was established in 1994 to provide educational information to patients, families, and health care providers. OncoLink is based at the University of Pennsylvania Cancer Center (UPCC) and currently serves between 1 and 2 million pages per month to over 385000 unique IP addresses.

**Figure 1:**
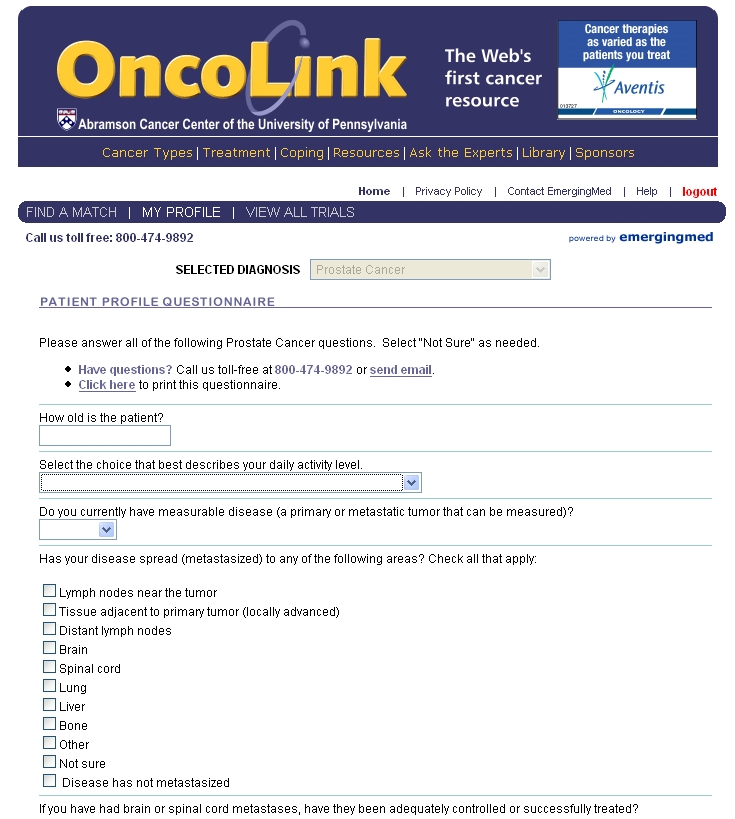
Screen shot of the OncoLink trial matching questionnaire

OncoLink launched the first broad cancer clinical trials matching resource on the Internet in January of 2002 in conjunction with EmergingMed (New York, NY). The resource was designed for patients to enter basic demographic data through a secure Internet connection (Figure 1). This information was then cross-matched with the inclusion and exclusion criteria of the protocol, and the patient was offered a matched list of potential trials for enrollment. For patients with an interest in obtaining more information or potentially enrolling in a matched clinical trial, appointments were facilitated with the principal investigators of the trials through personal communication.

Individuals interested in participating in the clinical trials matching service are encouraged to review the privacy policy of this resource. This details the purpose and use of the database as well as the use of the participant's personal information. By accepting entry into the matching service, participants agree only to allow matching to clinical trials approved by the institutional review board (IRB) and to facilitation of an appointment with the principal investigator should they meet enrollment criteria. This electronic consent is not a surrogate for study-specific consents, which must be obtained by the individual investigators after seeing and evaluating the patient personally. Each clinical trial in the database had specific IRB approval for inclusion in the matching service.

### Clinical Trials Matching Technical Details

**Figure 2:**
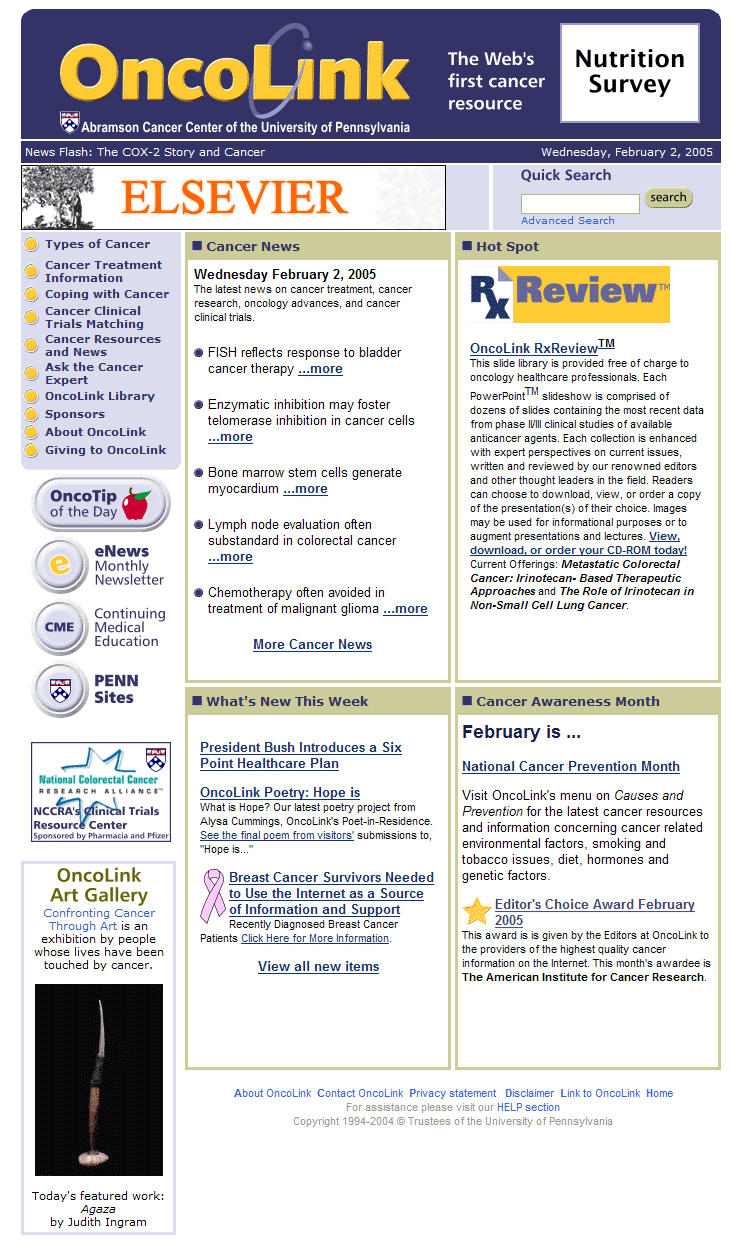
OncoLink home page

**Figure 3:**
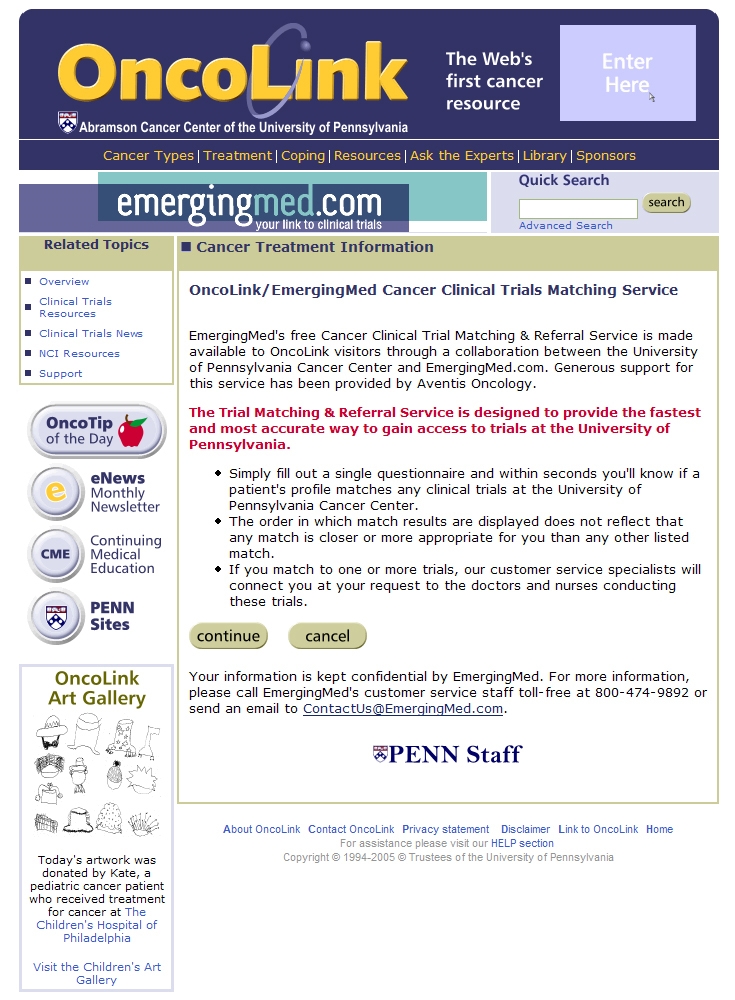
OncoLink subpage for entrance into the clinical trials area

**Figure 4:**
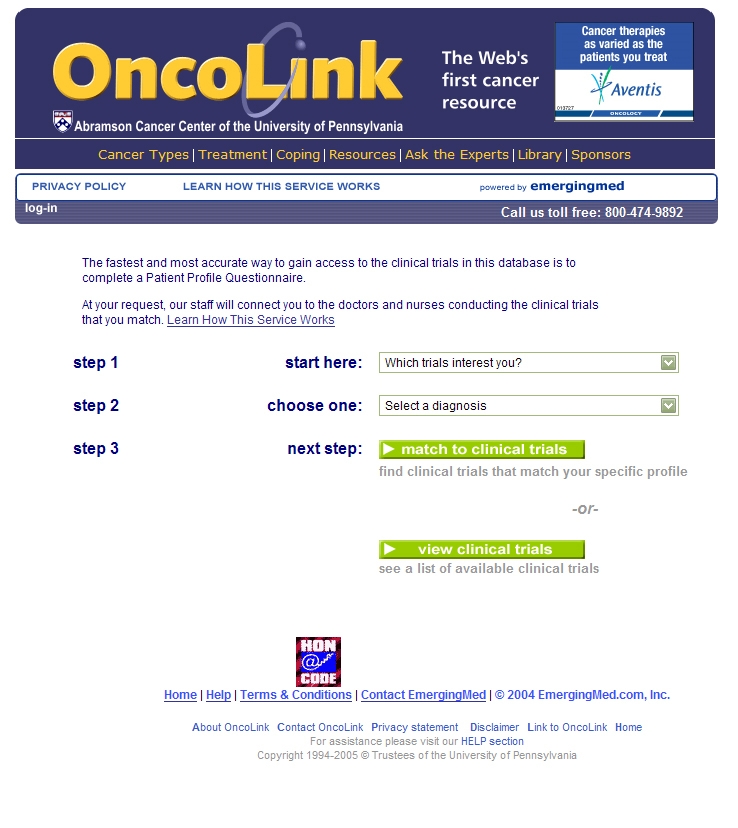
The first page of the matching service

Internet enrollment was conducted through the OncoLink website and data were held on a physically and electronically secure SQL server using secure socket layer (SSL) technology. In June 2004, the system was matching patients to 155 IRB-approved clinical trials at the UPCC. All currently open treatment trials are included in the system unless the principal investigator does not want the trial listed. There is no paid inclusion by the pharmaceutical industry to have a trial listed on the OncoLink service, and the matching is free from commercial bias. The initial pages of the matching service are shown in the screen shots in Figures 2-4. Additional pages are dependent on the specific cancer the individual is interested in matching. For those patients who do not live within the UPCC local area, a second-pass match is offered on a national level based on trials from the NCI PDQ^®^ database, participating cancer centers, and industry sponsored trials. In June 2004, this second-pass national match contained 2395 trials. This second-pass match, which is outside the scope of the OncoLink website and the University of Pennsylvania, may include trials whose inclusion is paid for by the pharmaceutical industry on the EmergingMed website. However, this paper only discusses the use of the OncoLink clinical trials service and website.

### Patient Questionnaire

The patient questionnaire contained queries regarding demographics, contact information, personal medical history, cancer diagnosis, and treatments to date. There was an internally validated questionnaire for each disease site that was developed based on the inclusion and exclusion criteria of all available trials. The questionnaire was designed to be interactive; therefore, individuals answered differing numbers of questions since follow-up questions were asked depending on previous responses. As some patients may not have known the specific details of their medical condition (eg, number of positive lymph nodes at time of resection), participants could score these answers as unknown. Data were only collected on patients that were actually matched to specific trials.

### Statistical Analysis

Statistical analysis was performed with SPSS software (version 9.0 for Windows; SPSS Inc, Chicago). Descriptive statistics were used to determine prevalence rates and demographic patterns of the individuals registered in the database. We did not keep track of patients who could not be matched.

## Results

Between January 2002 and April 2003, 627 patients submitted online applications and were matched for potential enrollment in cancer clinical trials through OncoLink. The mean age of the patient population was 56 years (range 18–88 years). Females were slightly younger than males (54 years vs 57 years; *P*≤ .05). Males represented 60% of the patient population. Over 90% of patients using this service were Caucasian. Of the 627 individuals eventually matched to clinical trials, online applications were initially submitted by 315 patients (50%), 293 family members (47%), 9 friends (1.5%), and 9 physicians (1.5%). For one patient (0.2%), it was unknown who entered the information into the system.

### Cancer Diagnosis of Participants

Table 1 shows the range of cancer diagnoses included in the population. The largest number of applications came from patients with colorectal cancer (13%), lung cancer (14%), melanoma (10%), and non-Hodgkin's lymphoma (9%). Stage IV disease was present in many patients using the Internet to match to clinical trials: breast cancer (50%), colorectal cancer (62%), lung cancer (41%), non-Hodgkin's lymphoma (41%), and head and neck cancer (45%).

**Table 1:** Distribution of patients by cancer diagnosis

**Diagnosis**	**Number (N = 627)**	**Percent**
Bladder	9	1.4
Brain	16	2.6
Breast	50	8.0
Cervical	12	1.9
Colorectal	83	13.2
Endometrial	4	0.6
Esophageal	6	1.0
Head and neck	22	3.5
Hodgkin's lymphoma	3	0.5
Kidney	12	1.9
Leukemia	17	2.7
Liver	13	2.1
Lung	90	14.4
Melanoma	60	9.6
Multiple myeloma	16	2.6
Non-Hodgkin's lymphoma	57	9.1
Ovarian	46	7.3
Pancreatic	40	6.4
Prostate	19	3.0
Rare tumors	16	2.6
Sarcoma	23	3.7
Stomach	12	1.9
Testicular	1	0.2

### Phase of Matched Trials

Table 2 shows the phase distribution of trials for those patients that applied for enrollment after review of their matches to specific trials. The median number of trials each patient was matched to was six.

**Table 2:** Phase of clinical trial for those who applied for enrollment after review of matched trials

**Phase**	**Number**	**Percent**
Pilot	1	0.4
I	63	22.0
I/II	28	9.8
II	96	33.7
III	48	16.8
Other	49	17.2

## Discussion

This study shows that patients are willing to use the Internet to find clinical trial information and enroll in trials for which they may be eligible. Motivated patients can research available trials and arrange a consultation appointment with the principal investigator of a specific trial. From the principal investigators' perspective, they are seeing a prescreened patient based on the inclusion and exclusion criteria of the trial who is already motivated to find a clinical trial that is right for them. This bypasses some of the major roadblocks that have been identified in the problem of clinical trials recruitment [3-12].

### Prevention Trials vs Treatment Trials

This matching service was developed based on the experience of OncoLink housing the National Colorectal Cancer Research Alliance (NCCRA) database for colorectal cancer prevention and treatment trials [24]. This database allows patients to answer a questionnaire detailing patient demographics, health parameters, and family history. After successful completion of the questionnaire, patients are entered into a large database that serves as a warehouse of patients interested in colorectal cancer prevention trials. The principal investigator of a trial may then search the database for participants that match the prevention trial parameters and contact the interested participant to discuss the trial. This type of database works well for prevention trials and genetic studies; however, it is not a good mechanism for cancer treatment trials. Patients with cancer need to make decisions on a tight timeline and need to know about potential trials in short order.

Thus, the current system was developed for immediate matching of patients with any cancer diagnosis with available trials. Although the matching system is completely automated, some personal interaction is still required, and appears to be desirable to the end users. Once patients are matched to clinical trials, they give permission to be contacted in order to facilitate an appointment with the clinical investigator. At the time of this phone contact, the patient's information is verified as is the trial information. The principal investigator (or designated nurse representative) is contacted to verify that the trial is still open and there have been no changes in the enrollment criteria prior to setting up the consultation. Any changes in the status of the trial are updated in the system, which serves as an internal validation.

### Cancer Diagnosis of Participants

The participants using this resource have a wide range of cancer diagnoses as shown in Table 1. Twenty-three different tumor types were classified in the system. The more common tumors, such as lung, colorectal, and breast, are seen frequently in patients using the system, as expected. Interestingly, patients with some less common malignancies, , such as melanoma, non-Hodgkin's lymphoma, and ovarian cancer, also showed a high utilization of the system. It may be that patients with these diagnoses are more actively searching the Internet for clinical trials information. However, this study was not designed to evaluate patients' reasons for using the Internet to identify clinical trials.

### Phase of Matched Trials

There was a broad listing of clinical trials to which the patients were matched (Table 2). There was no significant difference between the phases of trials which were matched with the exception of pilot studies. There were only a few pilot studies in the system at the time of this analysis, which most likely contributed to the low number of matches to this type of trial. The website is designed to match patients to all available trials without bias to any phase, sponsor, or specific trial design.

### Patient Enrollment by Surrogates

Family members entered 47% of the applications for patients enrolled in the system. It must be recognized that many patients rely on family and friends to obtain Internet-based information. A questionnaire study by Vordermark et al evaluated 139 German radiation oncology patients [25]. The Internet was used by 12% of patients to obtain information about their cancer, but an additional 15% received Internet-derived information about their cancer from friends or family members. This should be considered a conservative estimate since some patients (and family and friends) may not disclose their sources of information to physicians. Yakren et al analyzed the use of media information, including the Internet, among cancer patients and their companions at Memorial Sloan-Kettering Cancer Center [26]. Of the 443 individuals who returned completed surveys, 44% of the patients and 60% of the companions reported use of the Internet to obtain cancer-related information. The use of surrogates to obtain cancer clinical trials information and the high utilization of the system by family members have implications for the development of future versions of the software in order to ease the utilization of the system by these surrogates.

### Challenges of a Matching System

The Internet-based matching system requires a dedicated individual to keep an up-to-date and accurate listing of all trials, contact information, and inclusion and exclusion criteria. Although there is a national second-pass match in this system for those that cannot travel to the University of Pennsylvania, the upkeep of the basic data is outside the control of the resource.

There are some significant challenges for the establishment of a true national matching system for cancer clinical trials. The national infrastructure is not in place to accurately present clinical trials information from all institutions. No matter what computer program is utilized, the final product is dependent on correct and updated data from which it is derived. Most of the systems rely on listings from a variety of websites, most notably the National Cancer Institute (NCI). Unfortunately, much of this information is out of date, with incorrect contact information and trial listings (personal communication, October 2004). The principal investigator is often responsible for updating the system with any changes in contact information, posting of new trials, and removal of trials once they are closed. For those patients that do find a trial in which they are interested, many have difficulty contacting the investigator or ultimately find that the trial is not open for recruitment. This can be very frustrating for the end user.

### Recruitment of Minority Populations

Over 90% of the users of this clinical trials matching service were Caucasian. This underscores the importance of reaching other ethnic populations. Despite the potential benefits of this clinical trials matching service, there is a distinct risk of recruiting a biased population into the database. Historically, several subsets of patients have enrolled in clinical trials at an even lower rate than the general population. Minority enrollment has been relatively low, in part due to a lower willingness of African American patients to enroll in clinical trials [27-30]. Minorities and patients receiving their care from non-academic or community-based hospitals (eg, Veterans administration hospitals) also lag behind the general population with regards to Internet access and use [17,18]. Such a discrepancy can potentially bias the population registering through the Internet, subsequently biasing the population enrolling in clinical trials through the matching system. Minorities are more likely to cite distrust of the medical establishment as a reason for not enrolling in clinical trials [28,30-33]. Less then 10% of individuals who registered with the online matching system were minorities, which is lower than that seen for minority enrollment in cancer treatment trials at the NCI [34]. Enrollment of older patients has also lagged behind enrollment of other patients [5,35,36]. Older patients are less likely to have access to or utilize the Internet [17]. The lower rate of enrollment of older patients and minority patients limits the ability to generalize the results of many clinical trials. These populations appear to be underserved with regards to Internet usage.

### Conclusions

This is the first report of a broad Web-based cancer clinical trials matching service. This study shows that cancer patients are willing to use the Web to assist them in finding clinical trials. Strategies must be developed to ensure that underserved populations are included in clinical trials matching and recruitment services. As the data set matures, future reports will address recruitment to specific clinical trials.

## Abbreviations

HIV: human immunodeficiency virus
NCI PDQ database: National Cancer Institute Physician Data Query database
SQL: structured query language
